# High level *MYCN* amplification and distinct methylation signature define an aggressive subtype of spinal cord ependymoma

**DOI:** 10.1186/s40478-020-00973-y

**Published:** 2020-07-08

**Authors:** Mark Raffeld, Zied Abdullaev, Svetlana D. Pack, Liqiang Xi, Sushma Nagaraj, Nicole Briceno, Elizabeth Vera, Stefania Pittaluga, Osorio Lopes Abath Neto, Martha Quezado, Kenneth Aldape, Terri S. Armstrong, Mark R. Gilbert

**Affiliations:** 1 Laboratory of Pathology, National Cancer Institute, National Institutes of Health, Bethesda, MD USA; 2 Neuro-Oncology Branch, National Cancer Institute, National Institutes of Health, Bethesda, MD USA

**Keywords:** Spinal cord ependymoma, *MYCN* amplification, Next generation sequencing, Methylation classifier

## Abstract

We report a novel group of clinically aggressive spinal cord ependymomas characterized by Grade III histology, *MYCN* amplification, an absence of *NF2* alterations or other recurrent pathogenic mutations, and a unique methylation classifier profile. Seven cases were found to have *MYCN* amplification in the course of routine mutational profiling of 552 patients with central nervous system tumors between December 2016 and July of 2019 and an eighth patient was identified from an unrelated set of cases. Methylation array analysis revealed that none of the 8 cases clustered with any of the nine previously described ependymoma methylation subgroups, and 7 of 8 formed their own tight unique cluster. Histologically all cases showed grade III features, and all demonstrated aggressive clinical behavior. These findings are presented in the context of data from three other studies describing similar cases. Therefore, a combined total of 27 *MYCN* amplified spinal cord ependymoma cases have now been reported in the literature, warranting their consideration as a distinctive subtype of spinal cord ependymoma (SP-EPN-MYCN) with their unique molecular characteristics and aggressive clinical behavior.

## Introduction

Ependymomas arising in the spinal cord are categorized histopathologically into three main subtypes: myxopapillary ependymomas (SP-MPE), subependymomas (SP-SE) and classical ependymomas (SP-EPN). The SP-MPE and the rare SP-SE generally have low grade features and are both considered Grade I tumors in the 2016 WHO classification of Tumours of the Central Nervous System [4]. Most classical SP-EPN show Grade II features, but a minority of cases have more aggressive appearing histology that may include increased cellularity, increased mitotic rate, microvascular proliferation and necrosis, and are classified as anaplastic or Grade III ependymomas. The distinction between Grade II and Grade III ependymomas can be difficult and subject to interpreter bias [4], however the clinical importance of this distinction is becoming increasingly clear as evidenced by a recent large epidemiological study of 1345 patients showing a much poorer overall 5 year survival in patients with Grade III tumors than patients with Grade II disease [14]. Furthermore, current NCCN guidelines recommend adjuvant radiotherapy in all Grade III cases, a recommendation that is not as strongly made for grade II tumors [7]. Thus, biomarkers that can assist in distinguishing aggressive ependymomas from indolent ependymomas are critically needed.

In contrast to their counterparts arising in the brain, spinal cord ependymomas (SP-EPN) show frequent inactivating mutations and/or loss of heterozygosity of the *NF2* gene [1, 3]. This alteration is found in both SP-EPN that occur in the setting of Neurofibromatosis Type II as well as in those that arise as de novo somatic mutations [1, 3, 10]. However, other than this characteristic mutation, clinical researchers have been singularly unsuccessful in identifying additional recurrent driver mutations, and until very recently there have been no biomarkers that separate well behaved classic spinal cord ependymomas from those that are likely to be aggressive.

The recent application of methylation array technology as a classification aid in central nervous system tumors has proven to be a very robust methodology for defining histopathologic and molecularly defined subtypes of central nervous system (CNS) tumors, including ependymomas [2, 9]. Nine distinct ependymoma subtypes were initially defined including 6 supraspinal subclasses, and 3 spinal cord subclasses corresponding to the three histopathologically defined spinal cord ependymoma subclasses, SP-MPE, SP-ME, and SP-EPN [9]. Notably, in the original 2015 study, SP-EPN were underrepresented, comprising only 21 of the 500 cases in that study, with only one histologically determined Grade III tumor included.

In this study, we report a novel group of SP-EPN that are characterized by anaplastic Grade III histology, *MYCN* amplification, an absence of *NF2* alterations, and a unique methylation classifier profile. These findings are presented in the context of data from two recent studies describing similar cases [5, 12], and a third older study describing two additional cases. In agreement with the proposal by Ghasemi et al. [5], we believe that these *MYCN* amplified ependymomas should be recognized as a distinctive type of spinal cord ependymoma (SP-EPN-MYCN) with their unique molecular characteristics and aggressive clinical behavior.

## Materials and methods

### Cases

Materials from 644 cases from 552 patients with a variety of CNS tumors were evaluated by either an in-house custom CNS cancer next generation sequencing (NGS) panel (Primary Brain Tumor Panel (PBTP) and/or the Oncomine Comprehensive Assay v3 (OCAv3)) between December 2016 and July 2019 as part of their routine diagnostic evaluation. All patients reported in this study were enrolled in an IRB approved natural history protocol (NCI 16-C-0151), with the exception of one patient who did not enroll but provided informed consent for the molecular studies. One additional sample was selected for this report from the Collaborative Ependymoma Research Network (CERN) repository [13], after it was found to have *MYCN* amplification in an independent analysis of a subset of this study cohort by our group.

### Histologic examination

All available slides and materials were obtained from the archives of the Laboratory of Pathology at the National Cancer Institute (Bethesda, MD), except for the single case from the CERN archives. Cases were reviewed by expert neuropathologists during the course of routine histopathological evaluation. A secondary review of the eight *MYCN* amplified tumors was performed by two expert neuropathologists (MMQ and KA) for this study.

### MYCN immunohistochemistry

MYCN immunohistochemistry was performed on a Ventana Benchmark Ultra immunostainer (Ventana Medical Systems, Tucson, AZ) on deparaffinized paraffin embedded tissue sections using rabbit monoclonal antibody N-Myc (D4B2Y) from Cell Signalling Technology (Danvers, MA) at a dilution of 1:200. Prior to antibody application, antigen retrieval was performed on machine with Ventana Ultra CCI Cell Conditioning buffer at 95 degrees for 64 min. Signal amplification and detection was carried out with Ventana Amplification Kit and the Ventana ultraView DAB Detection kit.

### Next generation sequencing and nucleic acid extraction

Genomic DNA and RNA was extracted from 5 μm sections of formalin fixed paraffin embedded tissue sections using the AllPrep DNA/RNA FFPE Kit (Qiagen, Hilden, Germany). A portion of the DNA was set aside for methylation analysis as described below. Next generation sequencing was performed using either a custom amplicon based brain tumor specific panel (PBTP) or the Oncomine Comprehensive Assay v3 (OCAv3)(Thermo Fisher Scientific, Waltham, MA). The OCAv3 is a commercial amplicon-based panel and its content can be found on the manufacturer’s website (https://www.thermofisher.com/order/catalog/product/A35805#/A35805). The PBTP is a custom amplicon-based panel containing 927 PCR primer pairs designed to detect small nucleotide variants in 56 genes, copy number variants (CNVs) in 21 genes, and fusions between 25 gene pairs that have been associated with CNS cancer pathogenesis (Supplemental Table S1). For each panel, libraries were generated from genomic DNA and cDNA (Thermo Fisher Scientific, Waltham, MA), using AmpliSeq technology, pooled and then subjected to next generation sequencing using an Ion S5™ XL Sequencing System (Thermo Fisher Scientific). Signal processing, base calling, and alignment to the GRCh37/hg19 human genome assembly were carried out using Torrent Suite™ software packages (Thermo Fisher Scientific). Variant annotation and interpretation were performed with Ion Reporter Software v.5.10 (Thermo Fisher Scientific). All variants were manually reviewed by visualizing the raw sequencing read alignments using the Integrative Genomics Viewer (Broad Institute, Cambridge, MA). Final interpretation of variants was based on an integration of data from multiple bioinformatics databases and experimental and clinical data reported in the biomedical literature.

### Fluorescence in situ hybridization (FISH)

For FISH analysis, Vysis LSI N-MYC (2p24) SpectrumOrange Probe was obtained from Abbott Molecular (Des Plaines, IL, USA) and a centromeric probe CEP2 Aqua was purchased from Empire Genomics LLC (Williamsville, New York). The FISH experiments were performed as previously described [8]. In brief, 5 μm thick sections from formalin-fixed paraffin embedded tissue were incubated for 1 h at 60 °C to melt paraffin, and subsequently deparaffinized in xylene and rehydrated in a decreasing ethanol series finishing with PBS washes. Slides were steamed for 30 min in IHC-TEK epitope retrieval solution (IHC World, Ellicott City, MD, USA). The cytoplasm was removed by digestion using a solution of 100 mg/ml of Pepsin stock (Sigma, St. Louis, MO, USA) and 0.01 N HCL at 37 °C. The *MYCN* probe was pre-denatured at 78 °C for 5 min in hybridization buffer and applied to the slides. Co-denaturation was performed on the Thermobrite System (Abbott Molecular, Des Plaines IL, USA) at 73 °C for 5 min, followed by cooling to 37 °C and transfer to a humid chamber for overnight hybridization in a 37 °C oven. The slides were then washed in 0.4 x SSC (K.D Medical, Columbia, MD, USA)/0.3% Tween20 (Sigma, St. Louis, MO, USA) at 72 °C for 2 min followed by immersion for 1 min in 2 x SSC (K.D Medical, Columbia, MD, USA)/ 0.1% Tween20 (Sigma, St. Louis, MO, USA) at room temperature. The slides were counterstained, mounted with DAPI/Antifade (Vector Laboratories, Burlingame, CA) and analyzed on the BioView Duet-3 fluorescent scanning station using 63X-oil objective and DAPI/FITC/Rhodamine single band pass filters (Semrock, Rochester, NY). At least, 60 tumor cell nuclei were scored for each specimen. The number of red signals > 6 copies per cell in > 10% of cells (cutoff) was defined as amplification.

### DNA methylation-based analysis and CNV analysis

Two hundred fifty ng of extracted DNA was subjected to bisulfite treatment using the EZ DNA Methylation Kit (Zymo, Irvine, CA) and further processed and hybridized to Infinium MethylationEPIC (850 k) arrays, according to manufacturer’s protocol (Illumina, San Diego, CA). The arrays were scanned using an Illumina iScan reader. Raw signal intensities from the generated IDAT-files were analyzed using the “Classifier” tool developed by developed by Capper et al. [2]. Copy number variation (CNV) profiles were calculated and displayed using the “conumee” package for R version 3.5.2 [6].

## Results

### Identification of *MYCN* amplification in patient cases

**Fig. 1:**
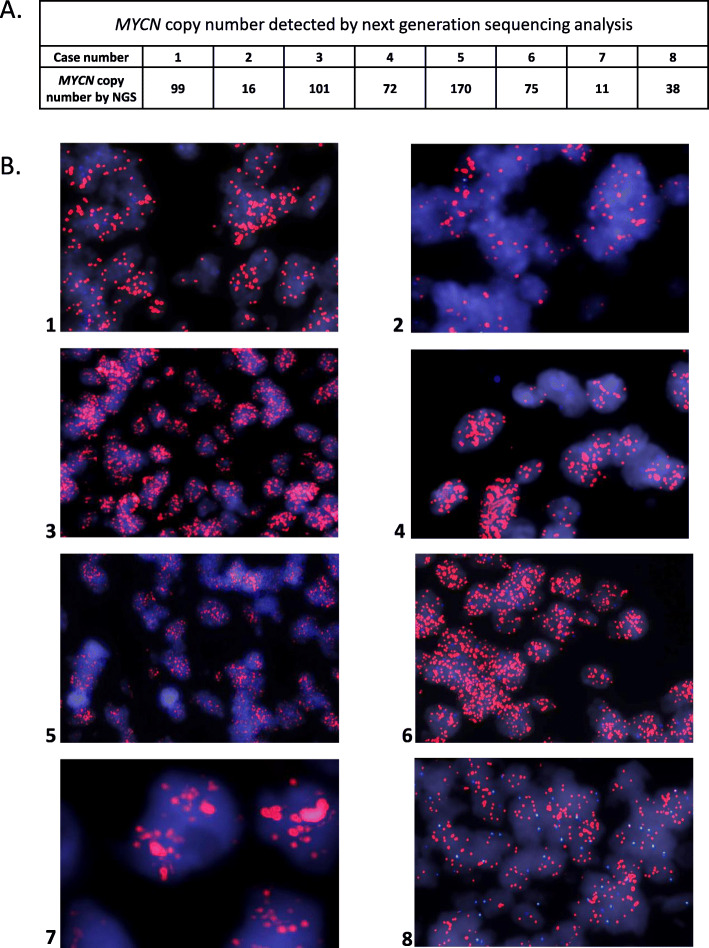
*MYCN* amplification detected by NGS and FISH studies. **a.***MYCN* copy number from next generation sequencing studies. All cases were interrogated by the OCAv3 NGS panel to obtain comparable data except for case 7 which was interrogated by the custom PBTP as there was insufficient material to reanalyze the case using the OCAv3 platform. **b.** FISH analysis of the 8 *MYCN* amplified cases showing high signal amplification in all cases

In the course of routine mutational profiling of 552 patients with CNS tumors reviewed in the Laboratory of Pathology, National Cancer Institute (USA) using a custom primary brain tumor specific next generation sequencing panel (PBTP) and/or the Oncomine Comprehensive Assay v3 (Thermo-Fisher Scientific), we identified 7 SP-EPN patients with high level *MYCN* amplification (Fig. 1a). An eighth case was identified in a separate CERN research study and is included in this report. With the exception of a pathogenic mutation in the *MAX* gene (c.179G > A, p.Arg60Gln) (Case 2), and a likely pathogenic *NOTCH2* variant (c.4622delA, p.Asn1541fs) (Case 4), no other significant single nucleotide variants or small indels, were detected among the eight cases, including *NF2* variants or evidence of copy loss in the *NF2* gene (Supplemental Table S2). *MYCN* amplification was confirmed by fluorescence in-situ hybridization in all 8 cases (Fig. 1b). None of 16 grade II spinal ependymomas or 26 classic ependymomas from the posterior fossa or a supratentorial location (14 Grade III EPN, 12 Grade II EPN) that were profiled in our laboratory displayed *MYCN* amplification.

### *MYCN* amplified ependymomas form a novel ependymal tumor methylation subgroup

**Fig. 2:**
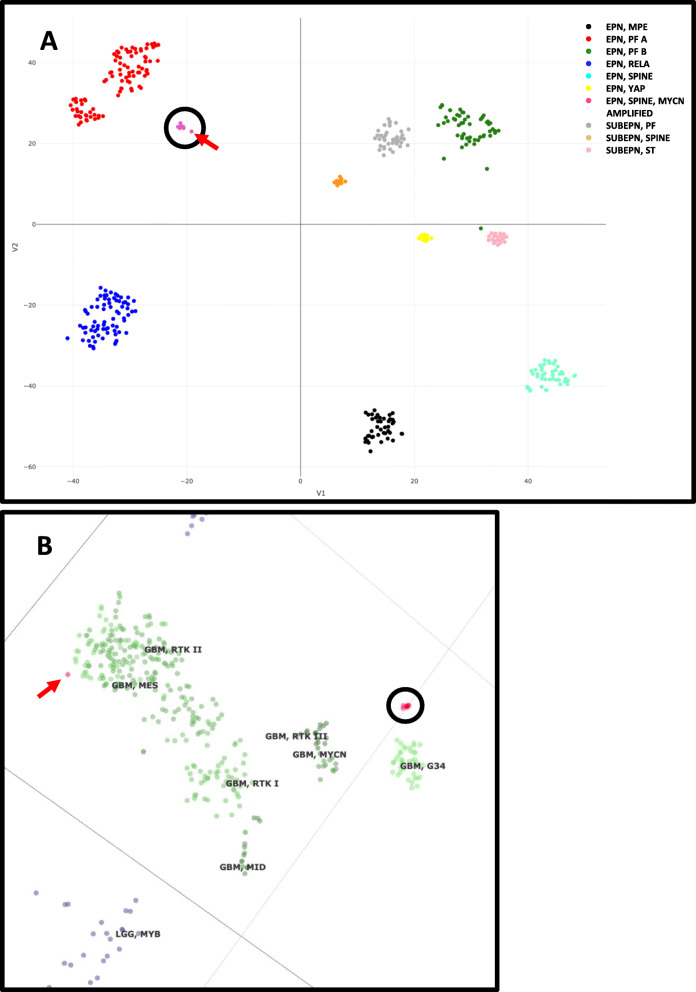
T-distributed stochastic neighbor embedding (t-SNE) plots of *MYCN* amplified cases. **a.** t-SNE plot demonstrating spatial separation of *MYCN* amplified ependymomas (black circle) from the previously described ependymoma subcategories in the DKFZ reference dataset. Seven of 8 cases show tight clustering. One case (case 4) is located outside the main cluster (red arrow). **b.** t-SNE plot demonstrating anomalous clustering of case 4 away from the main *MYCN* amplified cluster (black circle) and at the periphery of the mesenchymal glioblastoma cluster in DKFZ reference dataset (red arrow)

Methylation array analysis revealed that none of the eight *MYCN* amplified cases could be confidently classified within any of the 9 known ependymoma methylation subgroups, including those from the posterior fossa and the supratentorial region. Instead, all 8 cases formed their own distinct and novel cluster (Fig. 2a). Comparison to the complete methylation reference group continued to show *MYCN* amplified ependymomas clustering as a unique group, away from all other classified central nervous system tumors including *MYCN* amplified glioblastomas, with the exception of case 4 from the CERN archive that clustered at the periphery of the mesenchymal glioblastoma cases (Fig. 2b and Supplemental Figure S1). Copy number plots derived from the methylation arrays using the “conumee” package for R version 3.5.2 algorithm again confirmed *MYCN* amplification in all cases and revealed occasional additional copy number alterations (Supplemental Figure S2).

### Histopathology

**Fig. 3:**
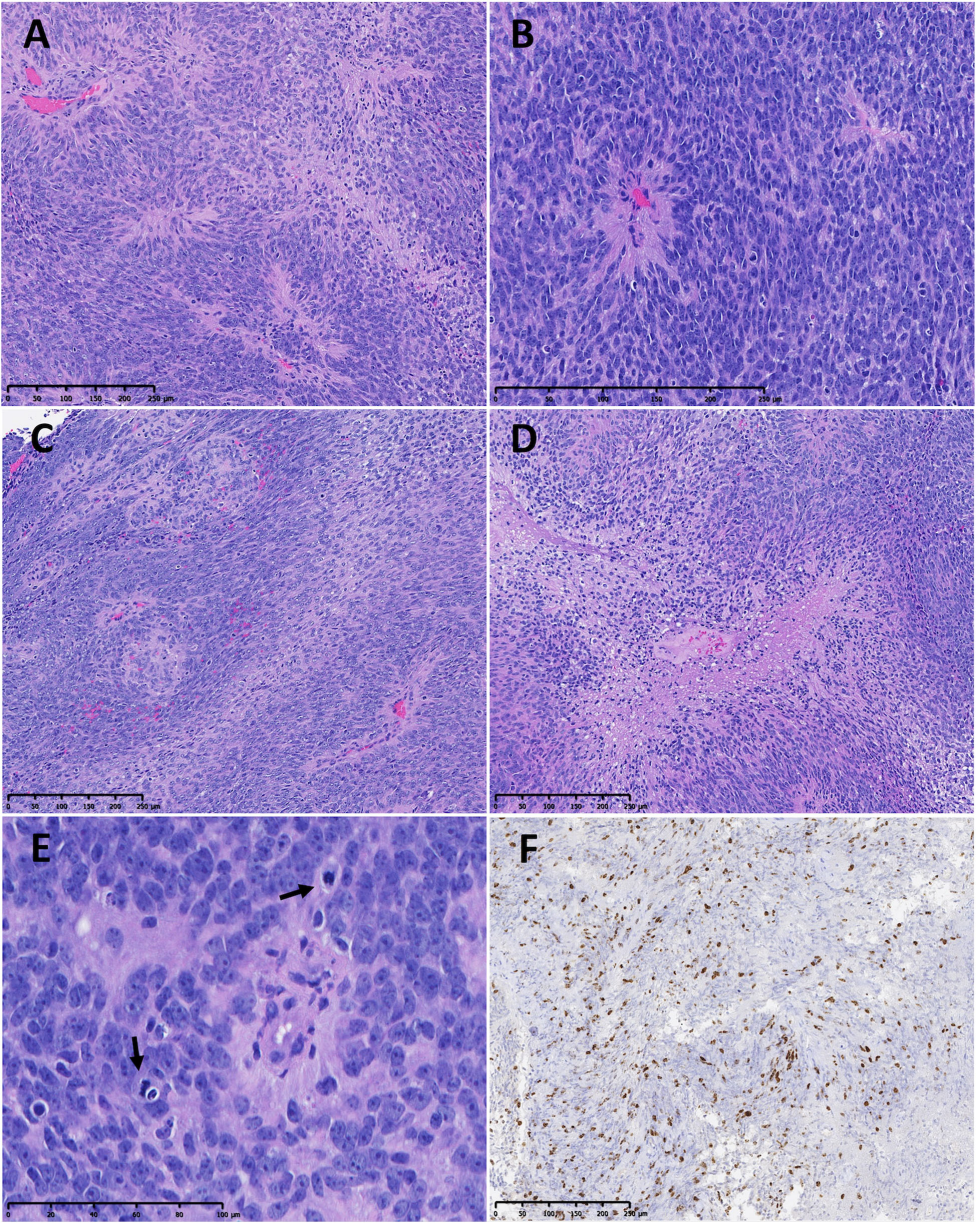
Histologic features of *MYCN* amplified spinal cord ependymomas, representative case 1 (A-E, H&E). **a, b.** Classical neuroectodermal ependymoma-like features showing typical cellular composition and pseudorosettes (**a.** 100x, **b.** 200x). **c, d, e.** Atypical high-grade associated features: **c.** vascular proliferation (100x), **d.** focal necrosis (100x), **e.** hyperchromatic tumor nuclei with prominent pink nucleoli and increased mitoses (400x) **f.** MIB1 immunostain demonstrating high proliferation rate (~ 20%) in case 5 (100x)

**Fig. 4:**
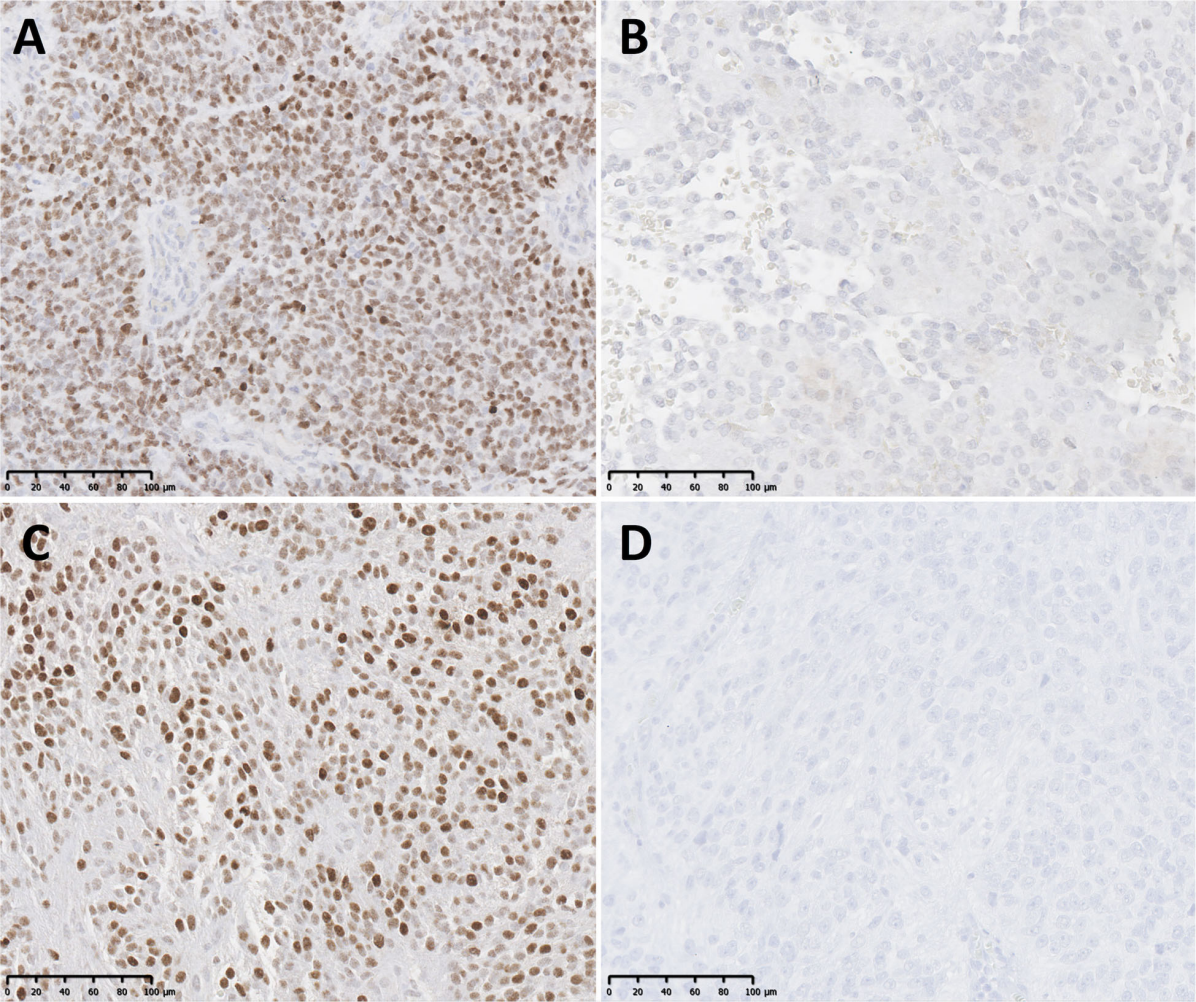
Representative examples of *MYCN* immunohistochemistry. **a, c.***MYCN* amplified SP-EPN. Cases 3 and 8. (200x). **b, d.** non-*MYCN* amplified classical grade II SP-EPNs (200x)

All eight cases with *MYCN* amplification were histologically diagnosed as Grade III (anaplastic) SP-EPN by two expert neuropathologists (KA and MMQ). The histological features of these cases are shown in Fig. 3 and Supplemental Figure S3 and are further detailed in Supplemental Table S3. Briefly, all lesions were glial in nature and contained perivascular pseudo-rosettes. Hypercellularity was a feature in all tumors. All but the CERN case (case 4), displayed marked cytological atypia with nuclear hyperchromasia and prominent pink nucleoli. True rosettes or tubules/canals were not appreciated. The majority of the cases exhibited geographic necrosis. Glomeruloid vascular proliferation was observed in all cases and was seen in many vascular profiles. Mitoses were easily encountered and mitotic activity was > 7 mitoses/10HPF in 6/8 cases. Where immunohistochemistry staining was available for review, the typical ependymoma profile with GFAP and EMA dot-like positivity was seen. MIB1 immunostaining was available in 1 of the studied cases (case 5), showing an estimated proliferative index of 20% (Fig. 3). Six cases were stained retrospectively for MYCN protein and all showed strong overexpression, while 6 classic Grade II spinal cord ependymomas were negative for MYCN protein expression (Fig. 4, Supplemental Figure S4, and Supplemental Table S3).

### Clinical characteristics

The median age at diagnosis of the eight patients was 35.5 years (range 24–52) and there was no gender preference (4 males, 4 females) (Table 1). We were able to review the diagnostic imaging for 5 of the 7 patients seen at the NIH. This enabled us to accurately determine the location of the primary tumor, whether intramedullary or extramedullary and whether there was imaging evidence of dissemination. Four of these five patients had extramedullary tumors and 3 had evidence of dissemination (Supplemental Figure S5). The one patient with intramedullary tumor did not have dissemination. Of the two patients for whom only medical records were available, one patient was reported to have only extramedullary tumor with dissemination and the remaining patient had dissemination, although the location of the primary tumor was not stated. All but two of the most recent cases (followed for 2 and 14 months, respectively) had at least 1 recurrence with one patient experiencing 12 recurrences. Two patients recurred in both a spinal cord and brain location during the course of their disease. This recurrence rate of 75% of patients compares to a rate of around 5% for classical SP-EPN [14]. Six patients remain alive with disease; 2 have died of progressive disease at 80 and 85 months following their diagnosis. None of the patients had a history of other cancers with the exception of case 1 who had had a previous diagnosis of basal cell carcinoma.

**Table 1:** Clinical characteristics of *MYCN* amplified cases

Case	Sex	Age at Diagnosis	Diagnosis	Location at Diagnosis	LMD at Diagnosis	Spine involvement	Recurrences	Location of recurrence	Followup	Disease Status	Prior Oncological Hx
1	M	52	A-EPN (grade III)	T, B	Yes	ID, EM	0	NA	2	Alive	Basal Cell Ca
2	F	24	A-EPN (grade III)	C, T, L	Yes	ID, EM	0	NA	14	Alive	None
3	F	30	A-EPN (grade III)	T	No	ID, IM	3	T	17	Alive	None
4^a^	M	36	A-EPN (grade III)	T	No	unknown	4	C, LS	55	Alive	None
5	F	37	A-EPN (grade III)	T	No	ID, EM	3	T, B	62	Alive	None
6	F	35	A-EPN (grade III)	C, T	No	ID, EM	2	T	63	Alive	None
7	M	52	A-EPN (grade III)	C, T, L	Yes	ID, EM	1	C, T, L	80	Deceased	None
8	M	29	A-EPN (grade III)	C	Yes	ID, EM	12	C, B	85	Deceased	None

^a^ Case from CERN archive

*Abbreviations*: *LMD* leptomeningeal disease, *T* thoracic, *B* brain, *C* cervical, *L* lumbar, *LS* lumbosacral, *ID* Intradural, *EM* extramedullary, *IM* Intramedullary

Five of the 8 patients received a variety of chemotherapeutic or biologic agents (Supplemental Tables S4 and S5). All 5 received at least 2 cycles of Temozolomide or Temozolomide plus Lapatinib, and 3 patients also received Bevacizumab or Bevacizumab plus Carboplatin during their disease course, either before or after a trial of Temozolomide. Neither of these agents or combinations provided long term control of their disease. One patient received multiple different treatments including Temozolomide, Bevacizumab, 5-Fluorouracil, Carboplatin, Nivolumab and Lapatinib, and Vorinostat without any apparent effect. An additional patient also received 4 cycles of Topotecan, Depocyt, Gleevac and Hydroxyurea, in addition to courses of Temozolomide, and VP-16/Carboplatin without disease control.

## Discussion

In this report we describe a novel group of SP-EPN characterized by high level *MYCN* amplification that was detected on routine next generation sequencing of cases reviewed at the Laboratory of Pathology of the National Cancer Institute (Bethesda, MD). These cases were all classified as Grade III ependymomas by histologic criteria, often involved multiple sites, presented in an extramedullary location, had frequent leptomeningeal involvement, and were characterized by multiple recurrences and aggressive clinical behavior. MYCN protein overexpression could be detected in all 6 cases that were tested.

In addition to having *MYCN* amplification, none of these cases showed *NF2* abnormalities that are characteristic of the majority of SP-EPN, and importantly, none clustered with any of the previously described nine ependymoma methylation subgroups, and instead formed their own distinct methylation cluster. Interestingly, the single case from the CERN archive appeared to cluster at the periphery of the mesenchymal glioblastoma methylation group when the entire DKFZ reference set of CNS tumors was included in the analysis when visualized in the t-SNE plot. Nonetheless, despite its close proximity to the mesenchymal GBM methylation cluster, the methylation classifier did not return this classification or any other classification with a calibrated score > 0.3. The reason for the outlier clustering of this anomalous case is unclear. Clustering anomalies such as this were not reported in the study by Ghasemi et al. [5].

*MYCN* amplified cases comprised 31% of all Grade II/III SP-EPN seen on our pathology service between December 2016 and July 2019 and included the majority of the Grade III SP-EPN cases (7/11) from the NCI series. Note that the additional 8th case from the CERN archives is not included in the above statistic. It should be noted that as a tertiary care center our case cohort is likely to be skewed toward more aggressive cases, and that larger epidemiological studies suggest that Grade III SP-EPN comprise only around 5% of the total [14]. The lack of *NF2* mutations strongly suggests that these cases do not represent transformed classical Grade II SP-EPN. Furthermore, grade II histology was not seen in our case cohort.

Two other studies of *MYCN* amplified SP-EPN have been recently reported [5, 12]. In the larger of the two [5], the authors identified 13 SP-EPN cases that did not cluster in any of the known EPN methylation subgroups. Copy number variation plots derived from the methylation arrays revealed *MYCN* amplification, which was confirmed by fluorescent in situ hybridization studies in 4/13 patients with available tissue. Unlike our case 4, these authors did not report any anomalous clustering in the vicinity of the mesenchymal glioblastoma methylation cluster. Similar to our cases, these 13 cases showed aggressive clinical behavior with multiple recurrences and treatments. In contrast to our cases that all showed Grade III histology, three of their 13 cases were histologically classified as Grade II SP-EPN. Interestingly one case, originally classified as Grade II SP-EPN, recurred as a Grade III SP-EPN. Whether this represents histologic heterogeneity in *MYCN*-amplified cases, sampling differences, differences in histologic interpretation, or true histologic transformation, is unclear. These authors also presented data showing that MYCN immunohistochemistry (IHC) was able to identify all amplified cases, including one with Grade II histology, suggesting that a simple IHC assay for MYCN protein expression could be used as a screening test for *MYCN* amplified cases, especially important in cases that have ambiguous histology. Two of the 13 patients tumors were studied by RNAseq and neither had evidence of *NF2* mutation.

The second study described 4 *MYCN* amplified SP-EPN, all classified as Grade III [12]. Detailed clinical data was available for two of the four patients, both of whom recurred following aggressive treatment with one alive after 15 months of follow-up, and the second succumbing to progressive disease that included cerebral metastasis 82 months after initial presentation. Of the remaining two patients one died 6 months after presentation of unknown causes, and the fourth patient was lost to follow-up. Two of the patients’ tissues were studied by an NGS panel that included *NF2*, and neither showed *NF2* alterations.

In addition to these recent well documented studies, two similar cases were reported previously by Schiel et al. in 2001 [11]. Both cases were located in the spinal cord and showed high level amplification of *MYCN* by both Comparative Genomic Hybridization and FISH. Interestingly, the first case presented as a Grade II SP-EPN and transformed into an anaplastic SP-EPN the following year, with subarachnoidal dissemination. The second patient presented as a Grade III tumor and also had multiple concurrent schwannomas raising the possibility that the patient may have had an *NF2* associated Grade III ependymoma. However, *NF2* sequencing or copy number analysis was not performed in this study.

Together with the current study, 27 cases of *MYCN* amplified spinal cord ependymomas have been reported, all with aggressive behavior, and with the majority showing Grade III morphology (Table 2). Given these preliminary clinical and molecular data, there are several important considerations. First, the finding of the *MYCN* amplification should be considered pathognomonic for a high-grade neoplasm irrespective of the histologic grading. Secondly, as demonstrated in this study and by Ghasemi et al. [5], MYCN screening by immunohistochemistry can be used as a surrogate for *MYCN* amplification and should be considered for all non-myxopapillary spinal cord ependymomas that show extramedullary involvement and leptomeningeal spread or unusually aggressive behavior. Finally, given the propensity for dissemination, there should be consideration of more extensive radiation fields, either whole spine or craniospinal treatment. Currently, there is insufficient information regarding chemotherapy treatment, but the unique molecular findings may provide the opportunity to develop a tumor specific therapy.

**Table 2:** Literature review of *MYCN* amplified cases

Study (Ref)	Year	Grade II SP-EPN	Grade III SP-EPN	Total cases
Scheil et al. [11]	2001	1	1	2
Ghasemi et al. [5]	2019	3	10	13
Swanson et al. [12]	2019	0	4	4
Raffeld et al	CURRENT	0	8	8

## Conclusions

In conclusion, we describe 8 new cases and review recent literature on a novel aggressive subgroup of SP-EPN characterized by *MYCN* amplification, lack of *NF2* or other recurrent mutations, anaplastic Grade III morphology and aggressive clinical behavior. Although the histologic appearance conforms to the classic features of ependymoma, these tumors present exclusively in the spinal cord and form their own unique cluster on methylation array analysis. This study supports and provides additional cases and genomic data for the proposal by Ghasemi et al. that the *MYCN* amplified spinal cord ependymomas (SP-EPN-*MYCN*) be considered as a unique clinicopathological category within the broader group of SP-EPN [5].

## Supplementary information

**Additional file 1 : Table S1.** Custom PBTP NGS panel gene content.

**Additional file 2 : Table S2.** Genomic alterations detected by NGS in *MYCN* amplified SP-EPN cases.

**Additional file 3 : Table S3.** Pathological characteristics of *MYCN* amplified SP-EPN cases.

**Additional file 4 : Table S4.** Overview of therapies received at diagnosis and recurrence.

**Additional file 5 : Table S5.** Chemotherapies and biotherapies administered.

**Additional file 6 : Figure S1.** T-distributed stochastic neighbor embedding (t-SNE) plot of *MYCN* amplified cases showing the *MYCN* amplified ependymoma cluster (thick red arrow) and single outlier case 4 (thin red arrow) in the context of the complete reference set from the DKFZ. Note the extreme distance from the major group of classical spinal cord ependymomas identified with a red circle at bottom of the figure.

**Additional file 7 : Figure S2.** Copy number profiles displayed using ‘conumee’ package confirming *MYCN* amplification in all cases (red arrows) and demonstrating 1 to 2 additional alterations in most cases, with the exception of case 3 that shows multiple alterations.

**Additional file 8 : Figure S3.** Histology of the 8 *MYCN* amplified cases. Panels A-H correspond to cases 1–8 in numerical order. All photomicrographs are H&E, 100X, and most show areas with typical perivascular pseudorosettes characteristic of ependymomas.

**Additional file 9 : Figure S4.** MYCN immunohistochemistry. MYCN immunohistochemistry was performed as described in the Materials and Methods. Panels A-F correspond to cases 1, 2, 3, 5, 6, and 8. All photomicrographs are 200X. Note strong positive staining in tumor nuclei, and the absence of staining in vascular structures.

**Additional file 10 : Figure S5.** T2 weighted sagittal images demonstrating a large extramedullary tumor in the high cervical region and evidence of extensive dissemination over the entire spinal cord. A. Cervical imaging. B. Thoracic imaging. Arrows highlight areas of intradural, extramedullary tumor. Lesion in cervical region likely to be the primary site of disease.

## Data Availability

The genomic datasets generated during this study will be archived in the dbGap database and are also available from the laboratory. All other datasets presented in this manuscript are available from the laboratory on reasonable request.

## Abbreviations

SP-EPN-MYCN: Spinal Cord Ependymoma, MYCN Amplified
WHO: World Health Organization
SP-MPE: Myxopapillary Ependymoma
SP-SE: Subependymoma
SP-EPN: Spinal Cord Ependymoma
NCCN: National Comprehensive Cancer Network
NGS: Next generation sequencing
PBTP: Primary brain tumor panel
OCAv3: Oncomine comprehensive assay v3
t-SNE: t- distributed stochastic neighbor embedding
IRB: Institutional review board
CERN: Collaborative Ependymoma Research Network
PCR: Polymerase chain reaction
CNV: Copy number variant
FISH: Fluorescence In Situ Hybridization
IHC: Immunohistochemistry
GFAP: Glial fibrillary acidic protein
EMA: Epithelial membrane antigen
DKFZ: Deutsches Krebsforschungszentrum (German Cancer Research Center)
