# Supplementary material for: High level MYCN amplification and distinct methylation signature define an aggressive subtype of spinal cord ependymoma
Source: Acta Neuropathol Commun. 2020 Jul 8;8:101. doi: 10.1186/s40478-020-00973-y (PMC7346356; doi:10.1186/s40478-020-00973-y)
Supplement: Supplementary file 1 — Additional file 1 : Table S1. Custom PBTP NGS panel gene content. [file 40478_2020_973_MOESM1_ESM.docx]

**Supplemental Table S1. CUSTOM PBTP GENE CONTENT**

**Small Nucleotide Variants**

| ACVR1 | AKT1 | ATRX | BRAF | CCND2 | CCND3 | CDK4 | CDK6 | CDKN2A |
| --- | --- | --- | --- | --- | --- | --- | --- | --- |
| CHEK1 | CHEK2 | CIC | CTNNB1 | DDX3X | EGFR | FGFR1 | FUBP1 | H3F3A |
| HIST1H3B | HIST1H3C | HRAS | IDH1 | IDH2 | IGF1R | KLF4 | KRAS | MDM2 |
| MET | MSH6 | MYC | MYCN | NF1 | NF2 | NOTCH1 | NRAS | NTRK1 |
| NTRK2 | NTRK3 | PARP1 | PDGFRA | PIK3CA | PIK3R1 | PPM1D | PTCH1 | PTEN |
| RB1 | SETD2 | SIRT2 | SMARCA4 | SMARCB1 | SMO | SOX2 | SUFU | TERT |
| TP53 | TRAF7 |  |  |  |  |  |  |  |

**Copy Number Variants**

| CCND2 | CCND3 | CDK4 | CDK6 | CDKN2A | EGFR | FGFR1 | IGF1R | MET |
| --- | --- | --- | --- | --- | --- | --- | --- | --- |
| MYC | MYCN | NTRK1 | NTRK2 | NTRK3 | PARP1 | PDGFRA | PPM1D | PTEN |
| SOX2 | SUFU | TRAF7 |  |  |  |  |  |  |

**Gene Fusions**

| BTBD1 : NTRK3 | C11orf95 : RELA | C11orf95 : YAP1 | CLCN6 : BRAF | EGFRvIII |
| --- | --- | --- | --- | --- |
| EGFR : PSPH | EGFR : SEPT14 | ETV6 : NTRK3 | FAM131B : BRAF | FGFR1 : TACC1 |
| FGFR3 : TACC3 | GNAI1 : BRAF | GOPC : ROS1 | KIAA1549 : BRAF | MKRN1 : BRAF |
| MYB : QKI | NAB2 : STAT6 | NACC2 : NTRK2 | QKI : NTRK2 | RELA : RELA |
| RNF130 : BRAF | SRGAP3 : RAF1 | TPM3 : NTRK1 | YAP1 : FAM118B | YAP1 : MAMLD1 |
