# Supplementary material for: High level MYCN amplification and distinct methylation signature define an aggressive subtype of spinal cord ependymoma
Source: Acta Neuropathol Commun. 2020 Jul 8;8:101. doi: 10.1186/s40478-020-00973-y (PMC7346356; doi:10.1186/s40478-020-00973-y)
Supplement: Supplementary file 4 — Additional file 4 : Table S4. Overview of therapies received at diagnosis and recurrence. [file 40478_2020_973_MOESM4_ESM.docx]

| **Case** | **Age at dx** | **Sex** | **Therapy at diagnosis** | **Recurrence** | **Number of recurrences** | **Therapy at recurrence** |
| --- | --- | --- | --- | --- | --- | --- |
| 1 | 52 | Male | Subtotal resection, craniospinal radiation | No | 0 | NA |
| 2 | 24 | Female | Biopsy, craniospinal radiation | No | 0 | NA |
| 3 | 30 | Female | Gross total resection, involved field radiation | Yes | 3 | Gross total resection, chemotherapy |
| 4 | 36 | Male | Gross total resection, involved field radiation | Yes | 4 | Chemotherapy, craniospinal radiation, chemotherapy, subtotal resection, chemo-therapy, subtotal resection |
| 5 | 37 | Female | Gross total resection, involved field radiation | Yes | 3 | Involved field radiation, resection (2) |
| 6 | 35 | Female | Subtotal resection, craniospinal radiation | Yes | 2 | Subtotal resection, chemotherapy (2) |
| 7 | 52 | Male | Surgery, craniospinal radiation | Yes | 1 | Resection (7), chemotherapy (2) |
| 8 | 29 | Male | Subtotal resection, craniospinal radiation | Yes | 12 | involved field radiation, chemotherapy (4), subtotal resection (2), involved field radiation, chemotherapy |

**Supplemental Table S4. Overview of therapies received at diagnosis and recurrence**
