# Supplementary material for: High level MYCN amplification and distinct methylation signature define an aggressive subtype of spinal cord ependymoma
Source: Acta Neuropathol Commun. 2020 Jul 8;8:101. doi: 10.1186/s40478-020-00973-y (PMC7346356; doi:10.1186/s40478-020-00973-y)
Supplement: Supplementary file 5 — Additional file 5 : Table S5. Chemotherapies and biotherapies administered. [file 40478_2020_973_MOESM5_ESM.docx]

**Supplemental Table S5. Chemotherapy and biotherapies administered**
