# Supplementary figures and images for: High level MYCN amplification and distinct methylation signature define an aggressive subtype of spinal cord ependymoma
Source: Acta Neuropathol Commun. 2020 Jul 8;8:101. doi: 10.1186/s40478-020-00973-y (PMC7346356; doi:10.1186/s40478-020-00973-y)

## Slide 1
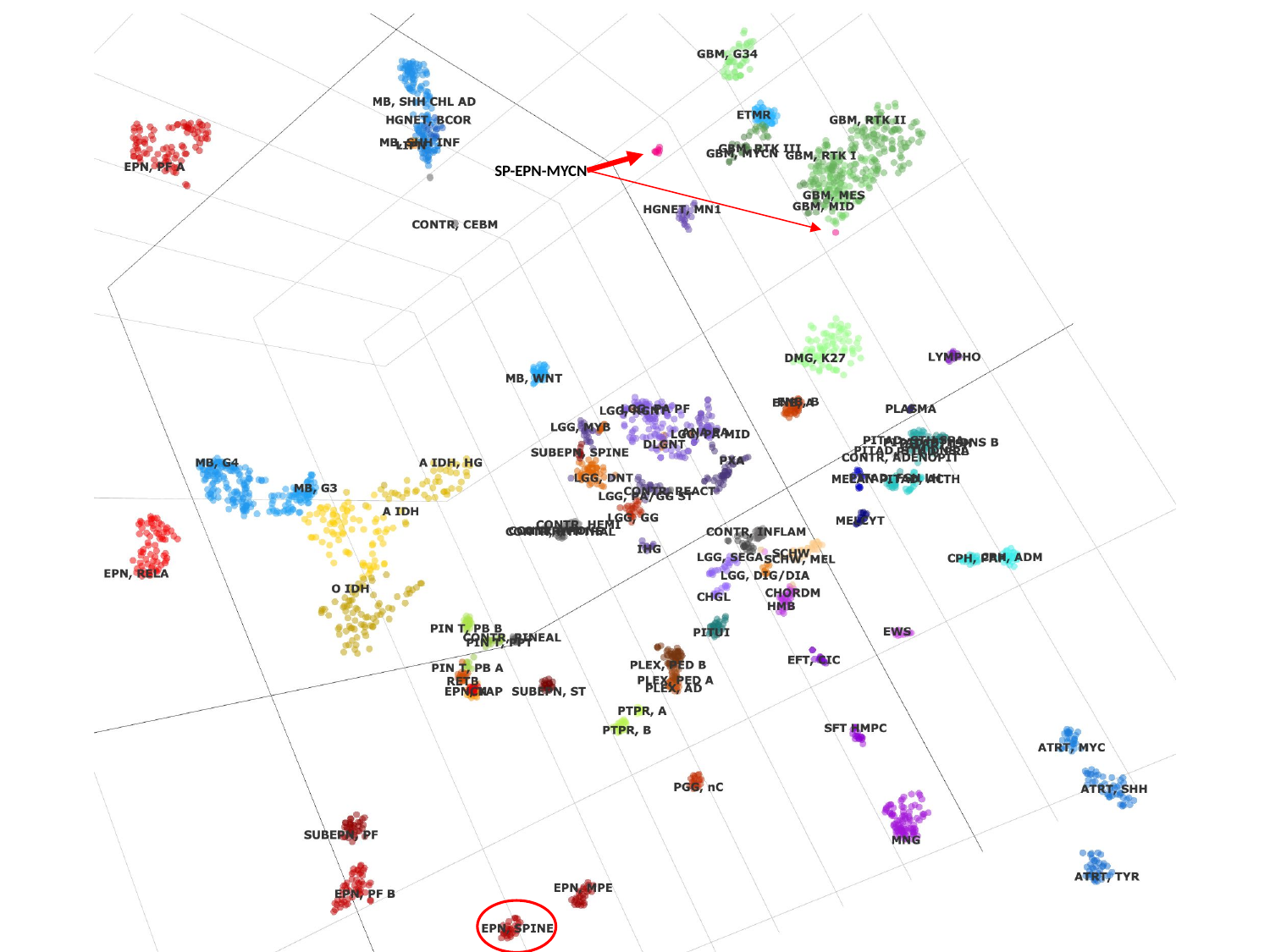

SP-EPN-MYCN

Supplement: Supplementary file 6 — Additional file 6 : Figure S1. T-distributed stochastic neighbor embedding (t-SNE) plot of MYCN amplified cases showing the MYCN amplified ependymoma cluster (thick red arrow) and single outlier case 4 (thin red arrow) in the context of the complete reference set from the DKFZ. Note the extreme distance from the major group of classical spinal cord ependymomas identified with a red circle at bottom of the figure. [file 40478_2020_973_MOESM6_ESM.pptx]

## Slide 1
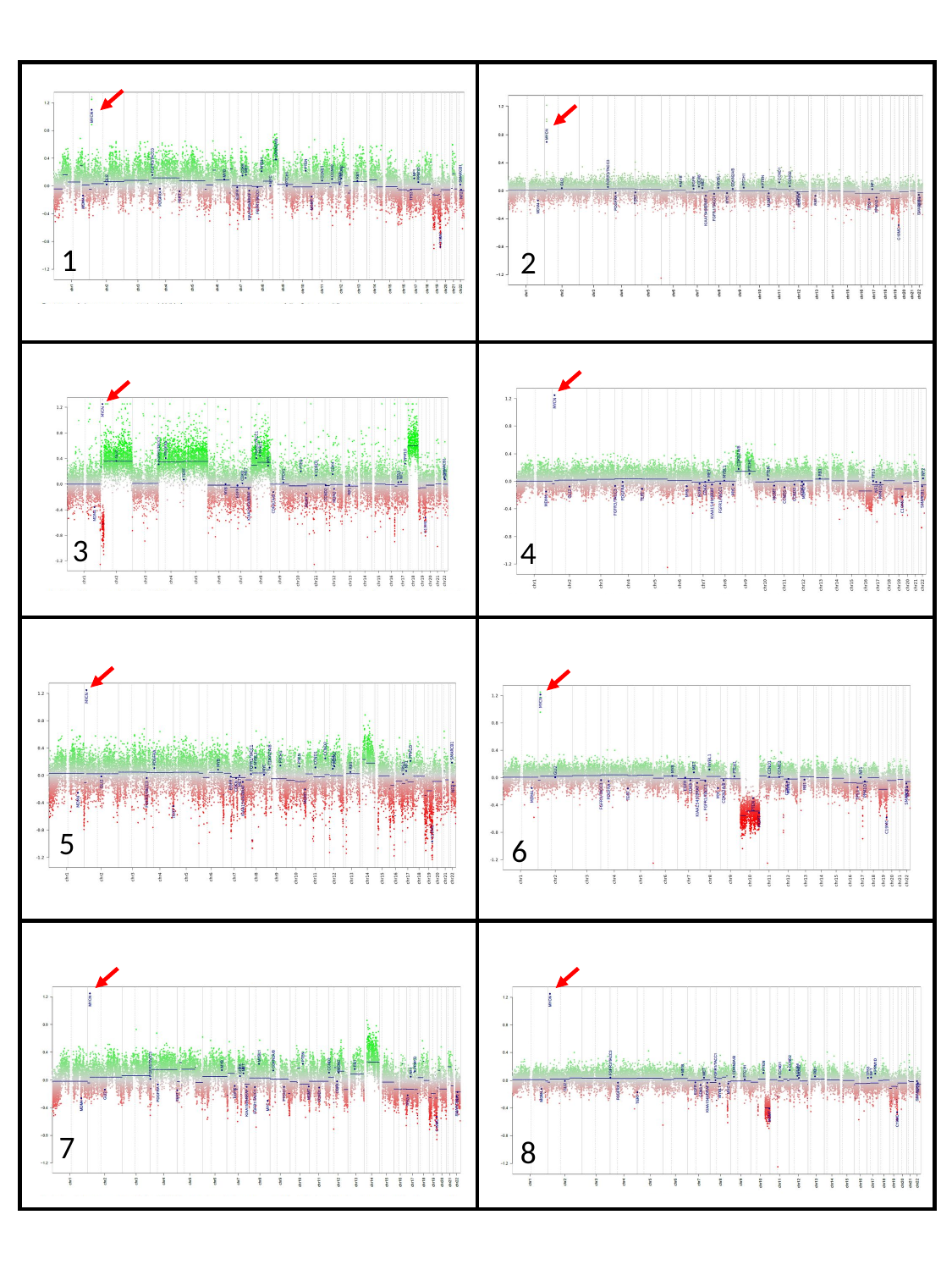

1
2
3
4
5
6
7
8

Supplement: Supplementary file 7 — Additional file 7 : Figure S2. Copy number profiles displayed using ‘conumee’ package confirming MYCN amplification in all cases (red arrows) and demonstrating 1 to 2 additional alterations in most cases, with the exception of case 3 that shows multiple alterations. [file 40478_2020_973_MOESM7_ESM.pptx]

## Slide 1
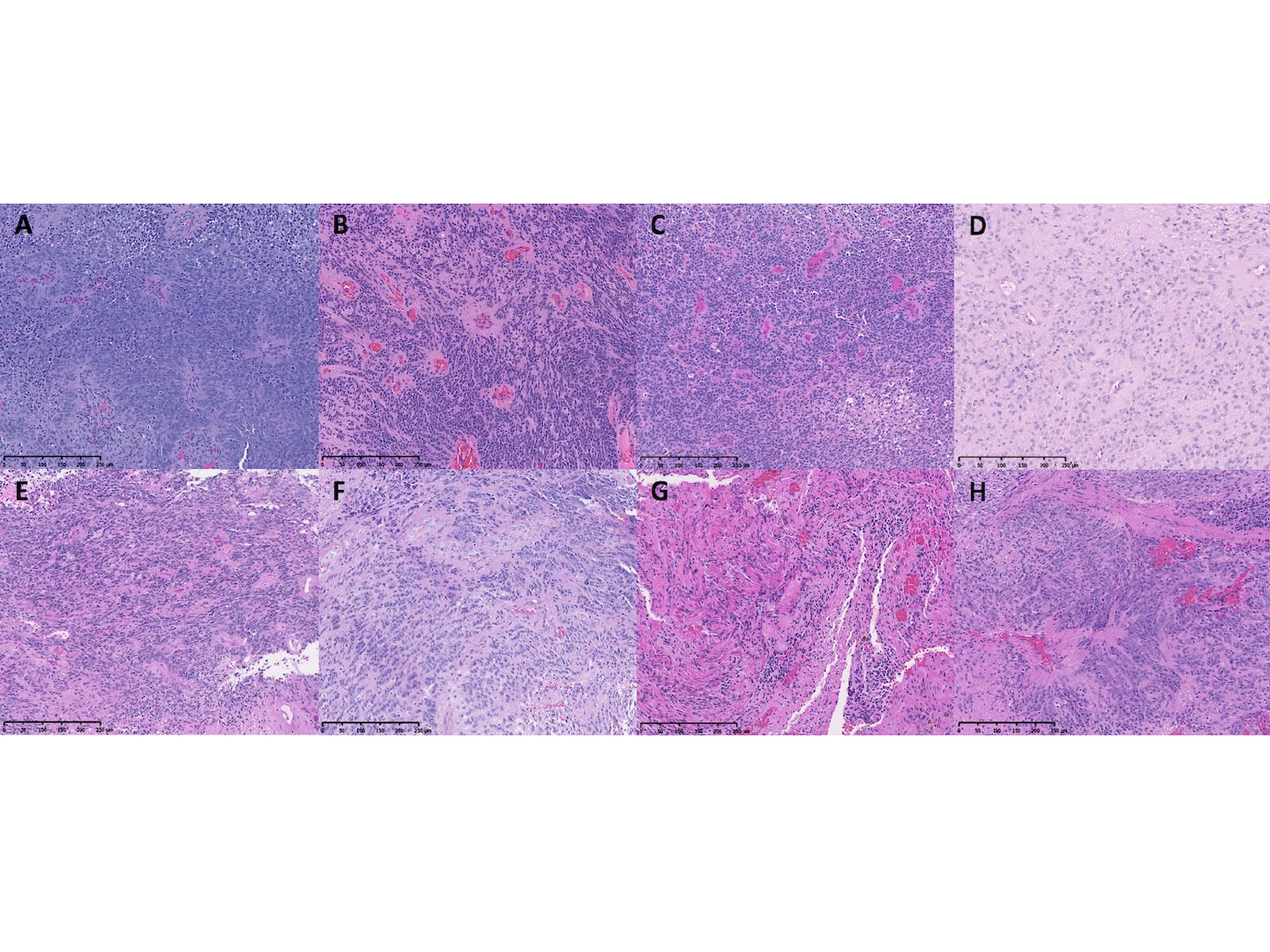

Supplement: Supplementary file 8 — Additional file 8 : Figure S3. Histology of the 8 MYCN amplified cases. Panels A-H correspond to cases 1–8 in numerical order. All photomicrographs are H&E, 100X, and most show areas with typical perivascular pseudorosettes characteristic of ependymomas. [file 40478_2020_973_MOESM8_ESM.pptx]

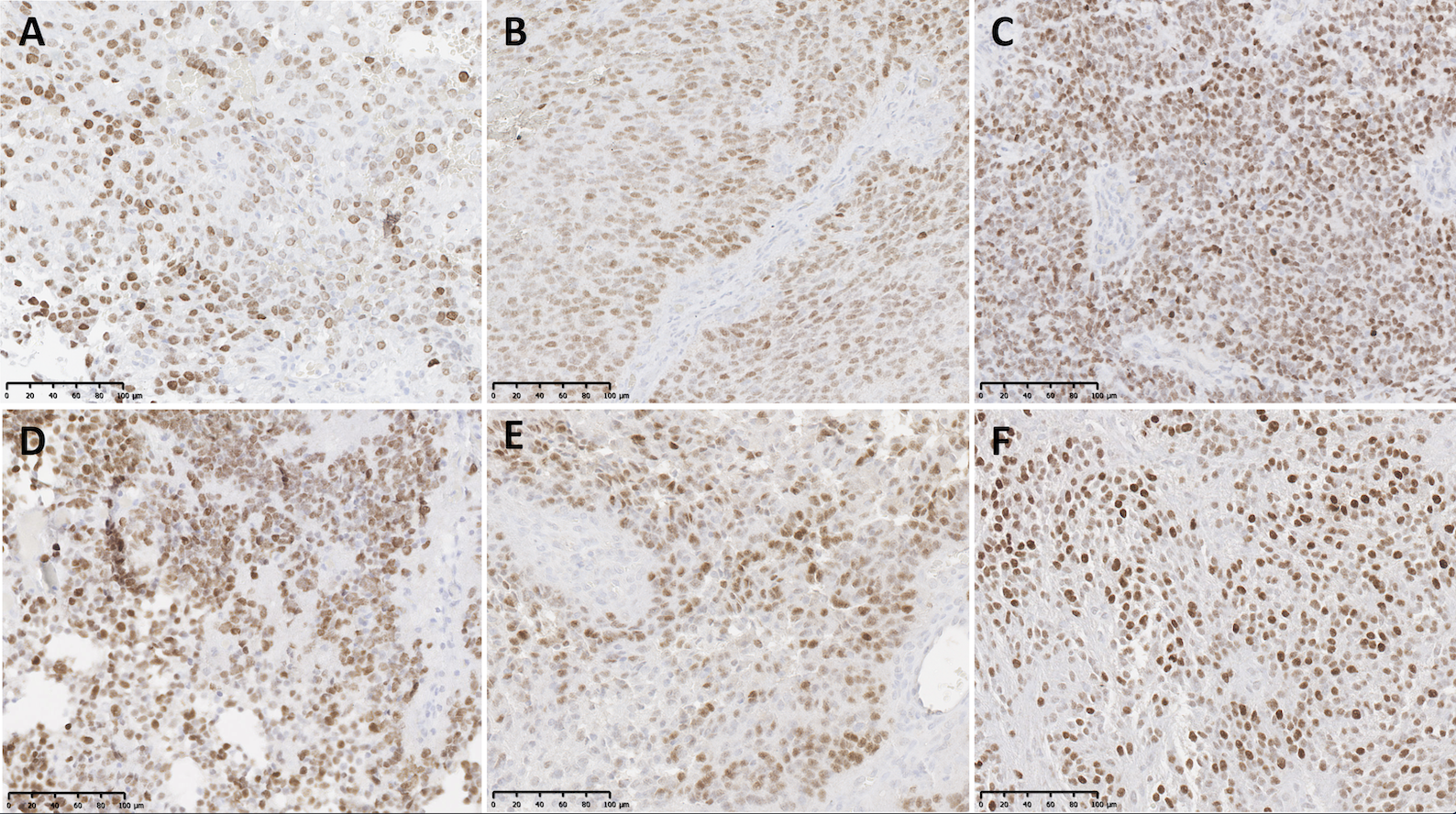

Supplement: Supplementary file 9 — Additional file 9 : Figure S4. MYCN immunohistochemistry. MYCN immunohistochemistry was performed as described in the Materials and Methods. Panels A-F correspond to cases 1, 2, 3, 5, 6, and 8. All photomicrographs are 200X. Note strong positive staining in tumor nuclei, and the absence of staining in vascular structures. [file 40478_2020_973_MOESM9_ESM.tiff]

## Slide 1
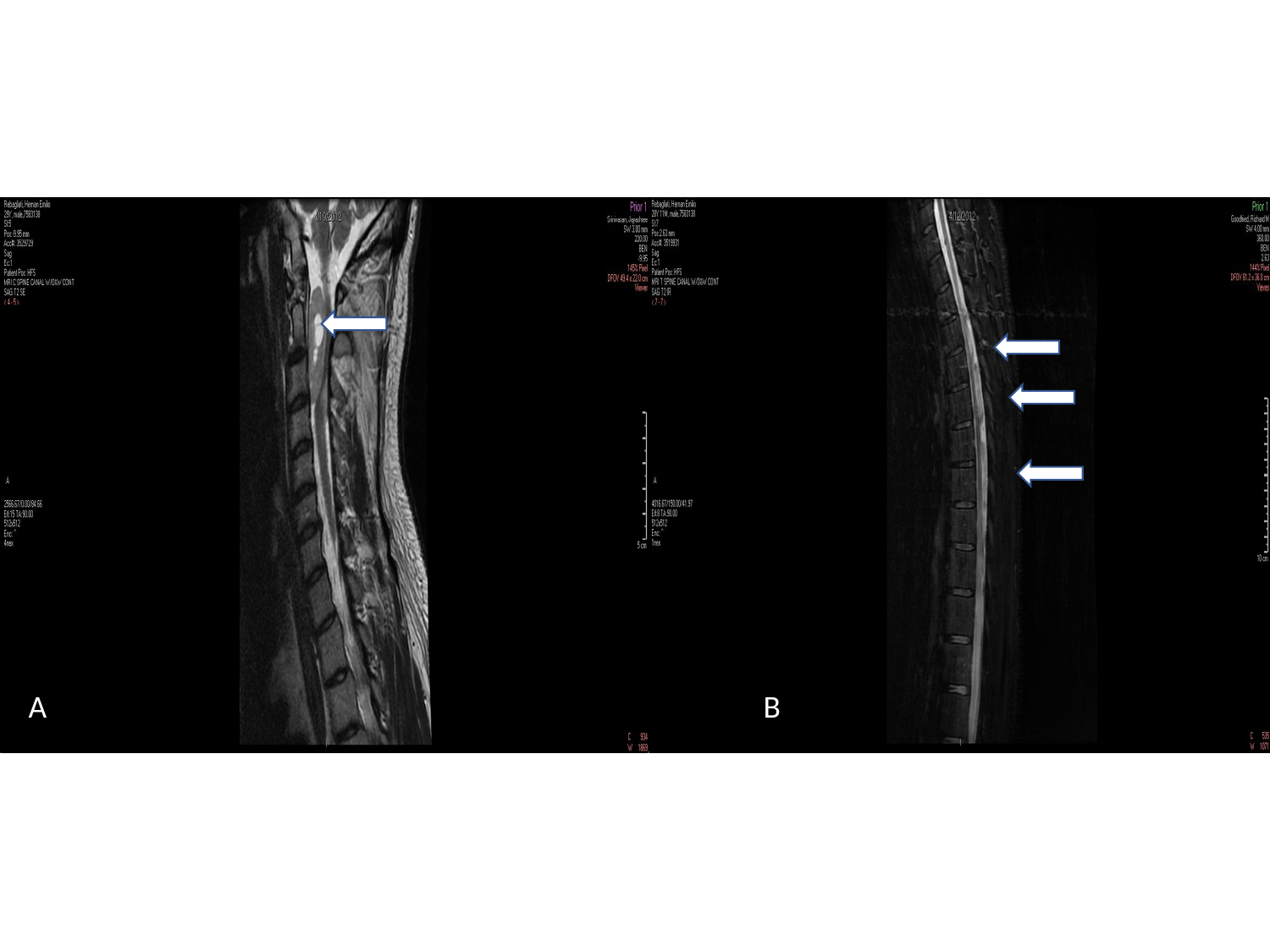

A
B

Supplement: Supplementary file 10 — Additional file 10 : Figure S5. T2 weighted sagittal images demonstrating a large extramedullary tumor in the high cervical region and evidence of extensive dissemination over the entire spinal cord. A. Cervical imaging. B. Thoracic imaging. Arrows highlight areas of intradural, extramedullary tumor. Lesion in cervical region likely to be the primary site of disease. [file 40478_2020_973_MOESM10_ESM.pptx]
